# Signet Cell in the Brain: A Case Report of Leptomeningeal Carcinomatosis as the Presenting Feature of Gastric Signet Cell Cancer

**DOI:** 10.7759/cureus.1085

**Published:** 2017-03-07

**Authors:** Saeed Ali, Muhammad Talha Khan, Evgeny A Idrisov, Aadil Maqsood, FNU Asad-Ur-Rahman, Khalid Abusaada

**Affiliations:** 1 Internal Medicine Residency, Florida Hospital Orlando; 2 Internal Medicine, Khyber Medical College; 3 Graduate Medical Education, Florida Hospital Orlando

**Keywords:** leptomeningeal carcinomatosis, signet ring cell cancer

## Abstract

Malignant infiltration of pia and arachnoid mater, referred to as leptomeningeal carcinomatosis (LMC), is a rare complication of gastric carcinoma. The most common underlying malignancy in patients with LMC are leukemia, breast cancer, lymphoma, and lung cancer.

We report a case of gastric adenocarcinoma that presented with LMC in the absence of overt gastrointestinal signs or symptoms. A 56-year-old Hispanic woman presented to the hospital with a three-week history of intermittent headaches and visual blurring. An initial brain imaging showed infarction in the distribution of right posterior inferior cerebellar artery (PICA) along with communicating hydrocephalus. She underwent ventriculoperitoneal (VP) shunt placement with improvement in her symptoms. Two months later she presented again with deterioration in her mental status. Imaging studies and cerebrospinal fluid (CSF) analysis confirmed the diagnosis of LMC. Further studies determined the primary tumor to be signet ring cell gastric adenocarcinoma. However, she did not have any preceding gastrointestinal symptoms. In light of the poor prognosis, the patient's family proceeded with comfort care measures.

Our case portrays a rare presentation of gastric adenocarcinoma with LMC without other distant organ metastatic involvement. It also illustrates the occult nature of gastric carcinoma and signifies the importance of neurologic assessment of patients, with or at risk of gastric carcinoma. ​It also raises a theoretical concern for VP shunt as a potential conduit of malignant cells from the abdomen to the central nervous system, which may serve as an important susbtrate for future research.

## Introduction

Malignant infiltration of pia and arachnoid mater, also known as leptomeningeal carcinomatosis (LMC), is a serious complication of certain malignancies. It is characterized by the diffuse spread of cancerous cells to leptomeninges via cerebrospinal fluid [1]. An autopsy study done by Grossman and Krabak revealed that up to five to eight percent of cancer patients may have LMC [2]. A significant proportion of these patients had asymptomatic microscopic disease, and therefore clinical diagnosis was reported in only two to four percent of patients. LMC is frequently detected in patients with leukemia, breast cancer, lymphoma, and lung cancer [2]. However, its incidence is only 0.14–0.24% among all gastric cancers [3]. LMC in gastric cancer can present as part of the initial clinical presentation or during late metastatic disease [1].

We present a case of gastric signet ring adenocarcinoma that was diagnosed as a result of workup for LMC. Informed consent was obtained from the patient for this study.

## Case presentation

A 56-year-old obese Hispanic woman presented to the hospital with a three-week history of intermittent headaches and visual loss. Her past medical history was significant for migraine headache and hypothyroidism.

Upon arrival, her vital signs were blood pressure (BP) of 136/85 mmHg, pulse of 85/minute, and respiratory rate of 15/minute. A complete physical examination including a thorough neurological examination did not show any focal cranial or peripheral neurologic deficit.

Initial laboratory studies revealed hemoglobin of 12.8 g/dL, white cell count of 12 x 10^3^/uL, and normal levels of serum electrolytes, urea, and creatinine. Magnetic resonance imaging (MRI) and magnetic resonance angiography (MRA) of the brain without contrast were done to rule out acute stroke, and they did not show any acute infarction or hemorrhage. On the second day of admission, she developed sudden deterioration in mental status with confusion, drowsiness, left lateral gaze palsy, and generalized stiffness. This prompted repeat MRI brain with and without contrast that showed infarction in the distribution of right posterior inferior cerebellar artery (PICA) along with communicating hydrocephalus. She was intubated and underwent lateral ventriculostomy placement to relieve the increased intracranial pressure. Cerebrospinal fluid (CSF) analysis was negative for infection while cytology was negative for atypical cells. She subsequently underwent ventriculoperitoneal (VP) shunt placement and was discharged to a rehabilitation facility after she showed clinical improvement.

**Figure 1:**
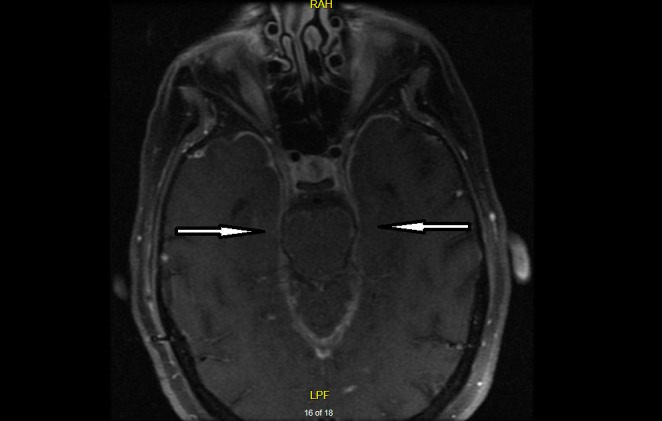
MRI of the brain with contrast showing widespread leptomeningeal enhancement (arrows) raising suspicion for leptomeningeal carcinomatosis.

**Figure 2:**
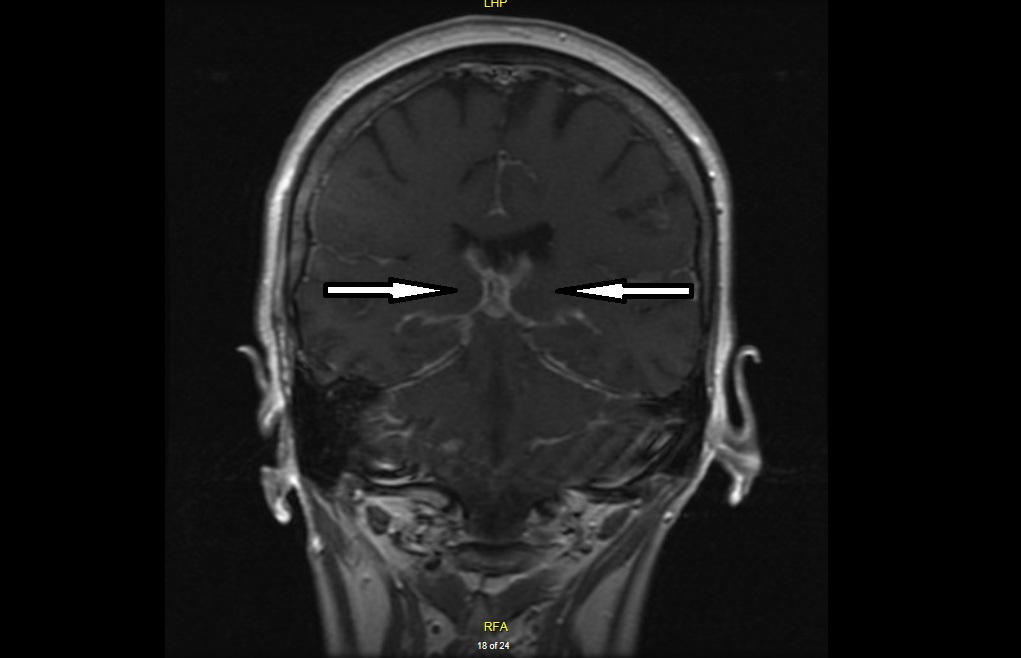
MRI of the brain with contrast (coronal section) showing widespread leptomeningeal enhancement (arrows) raising suspicion for leptomeningeal carcinomatosis.

Two months later, the patient presented again with worsening confusion, headache, nausea, and vomiting. An MRI of the brain with and without contrast revealed supratentorial and infratentorial leptomeningeal enhancement as seen in Figures 1-2. This finding raised concern for metastatic seeding of the meninges from a distant primary site of malignancy.

**Figure 3:**
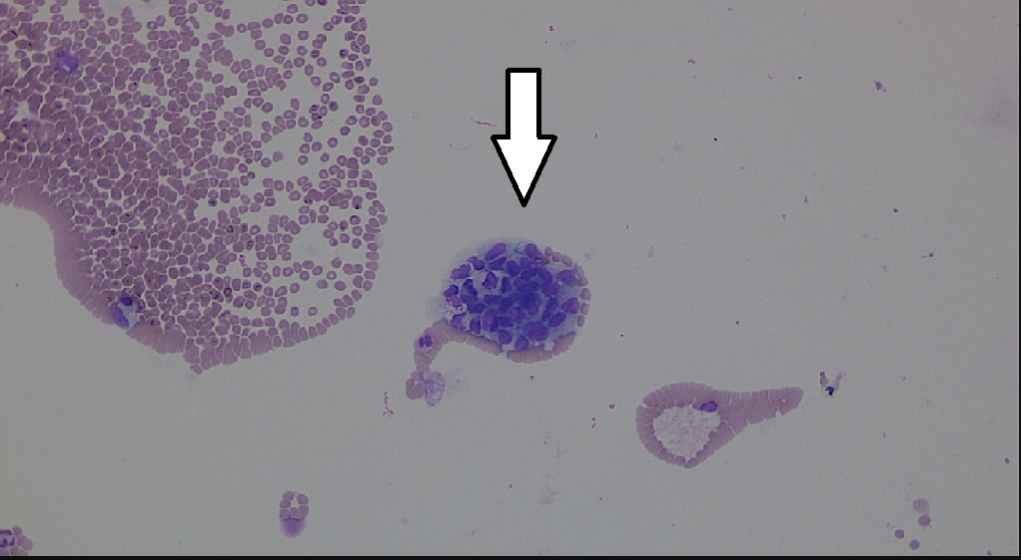
Clusters of atypical cells seen in CSF cytology.

The CSF analysis this time was significant for elevated protein (143 mg/dl), 4500 red blood cells, and 21 nucleated cells. The CSF cultures were negative for any bacterial or viral infection. The CSF cytology revealed atypical cells concerning for malignancy (Figure 3).

**Figure 4:**
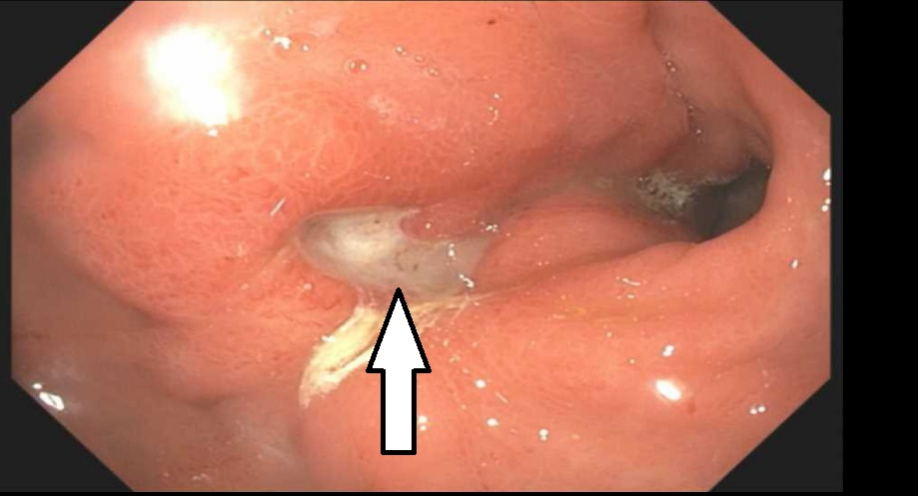
Endoscopic appearance of gastric ulcer (arrow).

An MRI of the cervical, thoracic, and lumbar spine with contrast showed leptomeningeal enhancement that further increased the suspicion for metastatic seeding. The patient did not have any chronic cough, shortness of breath, vaginal bleeding, abdominal pain, or chronic diarrhea that would suggest an obvious focus for underlying malignancy. Computed tomography (CT) imagings of the chest, abdomen, and pelvis with contrast were performed. The CT abdomen showed a thickened gastric lining with surrounding enhancement of the omentum. Based on the CT finding, an upper gastrointestinal (GI) endoscopic examination was done, that revealed a 15 mm pyloric ulcer with raised edges as seen in Figure 4. An endoscopic ultrasound showed diffuse thickening of the gastric wall with an 8 mm lymph node in the gastrohepatic ligament.

**Figure 5:**
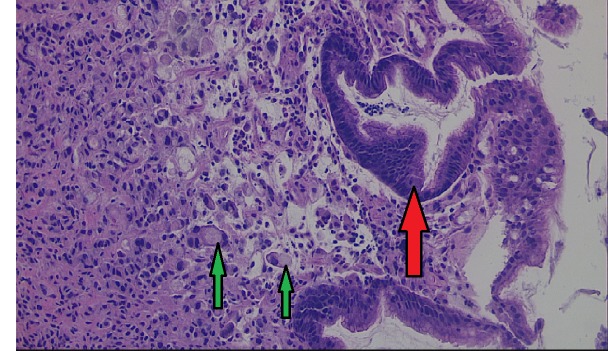
H&E staining showing normal gastric surface epithelium (red arrow). In some cells (green arrows) there are large mucin vacuoles in cytoplasm displacing nuclei and creating the characteristic signet ring appearance. H&E = hematoxylin and eosin.

A biopsy of the ulcer was suggestive of signet ring adenocarcinoma of the stomach (Figure 5).

Given the diffuse leptomeningeal spread and extensive intra-abdominal involvement of the tumor, the overall prognosis was poor. The patient's family opted for palliative and comfort measures only, and the patient was discharged home with hospice care.

## Discussion

The overall incidence of LMC for all cancers is reported to be two to four percent but is documented in up to eight percent from autopsy series [2]. Its incidence secondary to gastric carcinoma is approximately 0.14–0.24% [2-3].

LMC is frequently detected in patients with leukemia, breast cancer, lymphoma, and lung cancer. LMC secondary to gastric carcinoma is a very rare complication and in most cases, the histopathological type is signet ring cell carcinoma [4]. LMC usually develops in advanced gastric cancer where it has already metastasized widely to multiple organs including liver, lungs, and bone. The time lapse between diagnosing gastric cancer and development of neurological symptoms suggestive of LMC is approximately 12 months [4]. However, our patient's neurologic symptoms suggestive of the diagnosis of LMC predated the diagnosis of gastric carcinoma.

The  mechanism of spread of tumor cells to the leptomeninges is not clear. It is suggested that they can spread via arterial circulation, retrograde flow via Batson’s venous plexus, via perineural spaces, perivascular spaces, or lymphatics, and direct infiltration [5]. An intriguing fact in our patient was the placement of a VP shunt two months prior to the diagnosis of LMC. Whether the shunt could have been a route of spread of malignant cells from the peritoneum to the ventricular lining in the brain remains a question. It was also interesting that the patient did not have any substantial visceral spread of gastric cancer.

LMC can infiltrate cranial and/or spinal nerves, or it also can directly invade the brain or spinal cord leading to obstructive hydrocephalus and multiple neurological deficits. Thus, presentations can be diverse, and include nausea, vomiting, headache, diabetes insipidus, altered mental status, diplopia, facial numbness, hearing loss, loss of visual acuity, weakness of limbs, bowel or bladder dysfunction and a variety of other neurological deficits [4]. Our patient presented with confusion, headache, nausea, and vomiting.

Imaging studies are important for the diagnosis of LMC. The gadolinium-enhanced T1 sequences detect meningeal enhancement on MRI-brain that is characteristic of leptomeningeal disease; however, it is not a specific finding. Infectious and inflammatory conditions may show similar contrast enhancement. The sensitivity of contrast-enhanced MRI brain is reported to be between 65%–75% [6].

The gold standard test to diagnose LMC is CSF cytology. However, multiple samples may need to be obtained to increase its diagnostic yield. The sensitivity of the first CSF sampling is 54%. It increases to 91% with repeated sampling [1]. In our case, the second sample of CSF for cytology was diagnostic. Since the likelihood of detecting malignant cells increases in repeated samplings, one should not hesitate to repeat the test if the initial CSF analysis is negative and the clinical suspicion for LMC is high.

Treatment options in patients with LMC include steroids, radiotherapy, and intrathecal (IT) chemotherapy. The goal of the treatment is to improve neurological status and prolongation of survival. High dose steroids and radiotherapy provide symptomatic relief. There is a paucity of data for the duration of treatment for symptomatic relief. Chemotherapeutic agents for intrathecal chemotherapy include methotrexate (MTX), cytarabine (Ara-C), and thiotepa [1]. The median survival of LMC is around four to six months despite systemic or IT chemotherapy. Similarly, whether intrathecal CSF chemotherapy offers any advantage over systemic treatment has not been proven in randomized trials [3].

In a prospective study, Bokstein, et al. randomized patients with leptomeningeal disease to either radiotherapy, IT chemotherapy and systemic chemotherapy, or radiotherapy and systemic chemotherapy. The study showed that addition of IT chemotherapy did not improve overall survival whereas it increased early treatment-related complications [7].

The efficacy of single agent MTX versus combination MTX/Ara-C in IT chemotherapy has been studied by Kim, et al. They demonstrated improvement in cytology-negative conversion and median overall survival in the combination arm as compared to the MTX arm (38.5% vs. 13.8%, P = 0.03) and (18.6 weeks vs. 10.4 weeks, P = 0.02) [8]. In the annual American Society of Clinical Oncology meeting in 2009, Oh, et al. presented their data from a retrospective study demonstrating that cytology-negative conversion following IT chemotherapy predicted significantly prolonged survival [9].

Given the poor prognosis, palliative care is important. So far, there is a lack of evidence from randomized trials or observational studies to demonstrate superiority of intrathecal (IT) treatment compared to best palliative care.

## Conclusions

Gastric adenocarcinoma is a rare cause of leptomeningeal carcinomatosis. It is even rare to have LMC as a presenting feature of gastric cancer, without any significant abdominal symptoms and spread to other organs. Our case presentation is unique due to the recent placement of a VP shunt in the patient. This would serve as a new avenue of research to evaluate a novel potential route of cancer spread from the visceral organs to the central nervous system. Our case also embodies the occult nature of gastric cancer, and the need to thoroughly assess the neurological status of the patient with or at risk for gastric cancer.
